# CHILDHOOD HERPES ZOSTER: A CLUSTERING OF TEN CASES

**DOI:** 10.4103/0019-5154.48991

**Published:** 2009

**Authors:** Smitha Prabhu, H Sripathi, Sanjeev Gupta, Mukyaprana Prabhu

**Affiliations:** *From the Department of Skin and STD, Kasturba Medical College, Manipal, India*; 1 *From the Department of Skin and STD, Manipal College of Medical Sciences, Pokhara, Nepal, India*; 2 *From the Department of General Medicine, Kasturba Medical College, Manipal, India*

**Keywords:** *Childhood herpes zoster*, *human immunodeficiency virus*, *immunocompetent*

## Abstract

Herpes zoster occurs due to reactivation of the latent varicella zoster virus and is usually a disease of the elderly. Childhood herpes zoster is believed to be rare, though recent studies suggest increasing incidence in children. Here we report ten cases of childhood herpes zoster, seven of which occurred within a short span of six months, at a tertiary care level hospital in Pokhara, Nepal. Only three of the ten children reported previous history of varicella infection and none was immunized against varicella. Though childhood herpes zoster accounted for less than 1% of the total zoster cases in the past, recent reports show an increase in the number of cases in apparently healthy children. So far, no studies have been done linking childhood herpes zoster with HIV, though there are many studies linking it with other immunocompromised conditions.

## Introduction

Herpes zoster is a dermatomal viral infection, caused by the reactivation of the latent varicella zoster virus residing in infected dermatomes. Though a disease generally associated with old age, it is now increasingly occurring in otherwise normal children. Here we describe 10 cases of herpes zoster in children under the age of 14, only three of whom had underlying immunosuppression.

## Case Report

Patients number 1 and 2 were two HIV positive siblings, not on antiretroviral therapy, aged six and eight years, respectively [Table 1].

**Table 1:** Patient profile of childhood herpes zoster

Age	Sex	H/0 varicella	Immune suppression	Dermatome	Outcome
6	F	No	HIV	L T10-11	Infection
8	F	No	HIV	R L1-3	PHN
8	F	No	No	R T4-6	Good
10	F	Yes	No	L T12-L1	Good
6	M	No	No	L Opthal	Corneal opacity
11	F	Yes	ALL	L L1-2	Infection
7	M	No	No	R Trigem	Good
10	F	No	No	L T6,7	Good
13	M	No	No	R T8-10	Good
12	F	Yes	No	L T8-10	N/A

**Figure 1:**
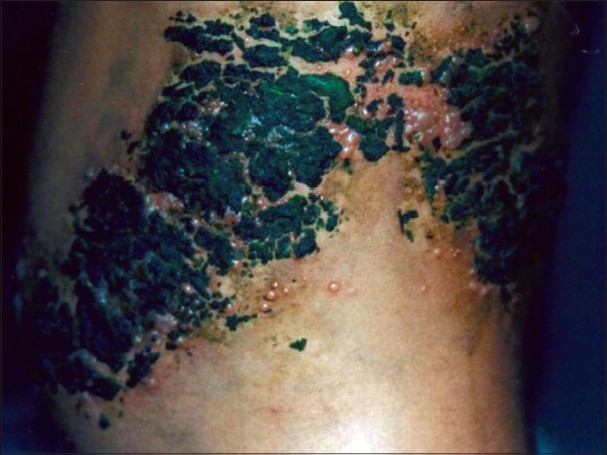
Case 3 - Grouped vesicles on erythematous base, over left thoracic (T10, 11) dermatomes. Herbal paste has been applied overneath

Case 3 was an eight-year-old healthy girl with thoracic zoster [Figure 1].

**Figure 2:**
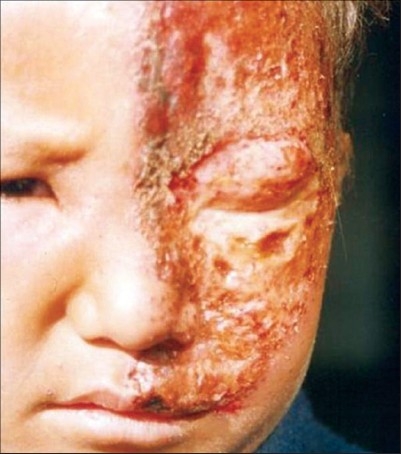
Grouped crusted erosions of ophthalmic zoster involving the left eye

Case 4 was a ten-year-old girl with left T12, L1 involvement, who gave a history of chicken pox six years back. Case 5 was a six-year-old immunocompetent boy, who presented to us late, with left ophthalmic zoster, along with weeping erosions and keratitis [Figure 2]. Case 6 was an 11-year-old girl, who had ulcerated grouped erosions on the left groin and pubis and who was receiving chemotherapy for acute leukemia,. Case 7 was a six-year-old-girl with right-sided trigeminal zoster, who showed good response to the treatment. Case 8 was a 10-year-old girl with left-sided thoracic zoster. Case 9 was a 13-year-old boy with right sided thoracic zoster. Case 10 was a 12-year-old girl with left-sided thoracic zoster.

None of the children was immunized against varicella infection and only three gave definite past history of varicella.

The investigations included Tzanck smear, which revealed multinucleate giant cells in three cases. Human immunodeficiency virus (HIV) ELISA (enzyme-linked-immunosorbent serologic assay) was negative in eight out of the 10 cases. Hemogram and peripheral smear was essentially normal in all the cases. Serum immunoglobulin levels, herpes simplex virus (HSV) antigen detection and viral culture were not done due to lack of facility. All the children were started on acyclovir tablets, with a dosage of 40mg/kg body weight, four times a day (up to a maximum dose of 800mg four times daily), for one week. Supportive measures included topical silver sulfadiazine cream, oral antibiotics and paracetamol.

## Discussion

All the 10 children were above five years of age. A majority of the children were females (seven cases, i.e. 70%). Only three children had definite history of previous varicella infection. None was immunized against varicella. Out of the 10 children, three were immunosuppressed – two siblings were HIV positive and one girl had acute leukemia. Thoracic dermatomal involvement was commonly observed (six cases, i.e. 60%). None had dissemination or multi-dermatomal involvement. Complications were noted in four children - one HIV positive girl had post herpetic neuralgia which lasted for six months; the other HIV positive girl and the girl with leukemia had secondary infection which responded to oral antibiotics. The child with ophthalmic zoster, who presented to us late in the course of the disease, developed corneal opacities and subsequent loss of vision in left eye.

Usually, primary varicella is a disease of childhood, whereas its reactivation infection, herpes zoster is encountered in the aged. The age adjusted incidence rate of herpes zoster is only 0.45 per 1000 persons in children below 14 years, but becomes as high as 4.5 per 1000 persons in those aged 75 and above. Herpes zoster in the elderly is associated with loss of varicella-zoster virus (VZV) specific cellular immunity, whereas in chemotherapy, suppression of cellular immunity *per se* occurs, and there is viral destruction of T cells in HIV infected individuals.[1]

Historically, childhood herpes zoster was thought to be an indicator for an underlying malignancy, especially acute lymphatic leukemia, whereas recent studies have shown no increase in the incidence of malignancy in children with herpes zoster. Approximately 3% of the pediatric zoster cases occur in children with malignancies.[2] Rising incidence of herpes zoster in otherwise healthy children may be due to acquiring primary varicella infection *in utero*, or in infancy, wherein the immunity is not fully developed. Vaccination with live attenuated virus may also contribute. Tereda *et al*. state that the immunological status at the time of acquiring the primary infection is the most important determinant in childhood herpes zoster. A low level of lymphocytes, natural killer (NK) cells and cytokines are seen in infants, along with virus-specific immunoglobulins, all of which may result in an inability to maintain the latency of VZV, leading to early appearance of zoster in children.[3]

A diagnosis of herpes zoster can be made at the bedside by a Tzanck smear preparation of scrapings from the floor of the vesicles, which will reveal multinucleated giant cells on direct microscopy, or by direct fluorescent antibody tests, presence of high or rising titers to VZV, or by culture studies.[4] It is imperative that herpes zoster be differentiated from zosteriform herpes simplex, which is more common in children, by direct fluorescent monoclonal antibody test or by detection of serum specific IgM by the indirect fluorescent antibody method. Ideally, in childhood herpes zosterx, lymphocyte counts, CD4/CD8 ratio, and serum immunoglobulin levels have also to be estimated to rule out undetected concurrent immunosuppression. The other common differentials for dermatomal vesicular eruptions in children are bullous impetigo and bullous insect bite reaction.

In general, the course of the disease is milder in children, the mean duration being 1-3 weeks. Though lesional pruritus and pain may be present, the incidence of post herpetic neuralgia is negligible. So far, almost all the reported series and isolated case reports have stressed upon the fact that childhood zoster is a relatively mild disease with negligible zoster associated pain, preherpetic neuralgia or other significant complications. The first line of therapy in childhood herpes zoster is oral acyclovir, given at a dose of 20-40mg/kg body weight, four times a day.[5] Patients with HIV infection are at a risk of developing severe illness from either varicella or zoster. Progressive primary varicella, a syndrome with persistent new lesion formation and visceral dissemination, may occur in HIV infected patients and may be life threatening. Though many studies have been done in adult HIV patients, so far there are no case reports of childhood HIV patients acquiring zoster.
